# The human sodium-dependent ascorbic acid transporters SLC23A1 and SLC23A2 do not mediate ascorbic acid release in the proximal renal epithelial cell

**DOI:** 10.1002/phy2.136

**Published:** 2013-11-07

**Authors:** Peter Eck, Oran Kwon, Shenglin Chen, Omar Mian, Mark Levine

**Affiliations:** 1 Department of Human Nutritional Sciences, University of ManitobaWinnipeg, Manitoba, Canada; 2 Department of Nutritional Science & Food Management, Ewha Womans UniversitySeoul, Republic of Korea; 3 Molecular and Clinical Nutrition Section, National Institute of Diabetes and Digestive and Kidney Diseases, National Institutes of HealthBethesda, 20892, Maryland; 4 Radiation Oncology, Johns Hopkins HospitalBaltimore, Maryland

**Keywords:** Ascorbic acid release, kidney, proximal tubule, SLC23A1, SLC23A2

## Abstract

Sodium-dependent ascorbic acid membrane transporters SLC23A1 and SLC23A2 mediate ascorbic acid (vitamin C) transport into cells. However, it is unknown how ascorbic acid undergoes cellular release, or efflux. We hypothesized that SLC23A1 and SLC23A2 could serve a dual role, mediating ascorbic acid cellular efflux as well as uptake. Renal reabsorption is required for maintaining systemic vitamin C concentrations. Because efflux from nephron cells is necessary for reabsorption, we studied whether SLC23A1 and SLC23A2 mediate efflux of ascorbic acid in the human renal nephron. We found high gene expression of *SLC23A1* but no expression of *SLC23A2* in the proximal convoluted and straight tubules of humans. These data rule out *SLC23A2* as the ascorbic acid release protein in the renal proximal tubular epithelia cell. We utilized a novel dual transporter-based *Xenopus laevis* oocyte system to investigate the function of the SLC23A1 protein, and found that no ascorbate release was mediated by SLC23A1. These findings were confirmed in mammalian cells overexpressing SLC23A1. Taken together, the data for SLC23A1 show that it too does not have a role in cellular release of ascorbic acid across the basolateral membrane of the proximal tubular epithelial cell, and that SLC23A1 alone is responsible for ascorbic acid uptake across the apical membrane. These findings reiterate the physiological importance of proper functioning of SLC23A1 in maintaining vitamin C levels for health and disease prevention. The ascorbate efflux mechanism in the proximal tubule of the kidney remains to be characterized.

## Introduction

Ascorbic acid (vitamin C) is indispensable for survival. Without it, the fatal deficiency disease scurvy occurs. Ascorbic acid is not synthesized by humans and other primates due to a loss of functional gulonolactone oxidase (EC 1.1.3.8), the terminal enzyme in the ascorbic acid biosynthesis pathway (Nishikimi et al. 1988; Padayatty et al. 2003). Ascorbic acid is a cofactor in many enzymatic hydroxylation reactions, and might be involved in redox homeostasis (Padayatty et al. 2003; Mandl et al. 2009). Suboptimal systemic levels of ascorbic acid might be coupled to common and complex diseases, such as birth complications, cancers, inflammatory, and cardiovascular syndromes (Padayatty et al. 2003). For all of these reasons, daily intakes of ascorbic acid are recommended for humans (National Research Council (USA) 1989).

Dietary ascorbic acid is readily absorbed in the gastrointestinal system, circulated in free form in the blood, transported against a concentration gradient into many tissues, freely filtered from plasma at the renal glomerulus, and reabsorbed in the kidney tubule system (Corpe et al. 2010). Two proteins responsible for ascorbic acid transport into cells have been identified: sodium-dependent ascorbic acid membrane transporter proteins SLC23A1 (SVCT1) and SLC23A2 (SVCT2) (Daruwala et al. 1999; Tsukaguchi et al. 1999; Wang et al. 1999, 2000).

SLC23A2 is found in the majority of cell types and mediates ascorbic acid transport across cell membranes with high affinity (K_m_ ∼10–20 μmol/L). Optimal activity is Na^+^, Ca^2+^, and Mg^2+^ dependent (Godoy et al. 2007). The physiological importance of SLC23A2 is demonstrated in knockout mouse studies. Global elimination of *Slc23a2* resulted in nearly undetectable ascorbate concentrations in all tissues measured, and *Slc23a2*^−/−^ knockout mice died within minutes of birth (Sotiriou et al. 2002). These data indicate that SLC23A2 is responsible for ascorbate tissue accumulation and is needed for survival (Sotiriou et al. 2002).

While SLC23A1 has a more limited distribution, it performs major transport functions by mediating ascorbic acid uptake into epithelial cells of the small intestine, liver and kidney (Maulen et al. 2003; Boyer et al. 2005; Lee et al. 2006; Luo et al. 2008; Varma et al. 2008), and is also expressed in some epithelia of the reproductive system and brain (Tsukaguchi et al. 1999). SLC23A1 transports ascorbic acid with moderate affinity (K_m_ ∼100–200 μmol/L) and high capacity, reflecting its role in intestinal absorption and renal reabsorption (Daruwala et al. 1999; Tsukaguchi et al. 1999; Corpe et al. 2005). Through global elimination in the mouse, it was found that SLC23A1 is necessary for renal reabsorption of ascorbic acid (Corpe et al. 2010). In the *slc23a1*^−/−^ mouse fractional ascorbic acid excretion increases up to 18-fold and has clearance similar to that of creatinine. The *slc23a1*^−/−^ mouse has low plasma and tissue concentrations and ∼50% perinatal mortality (Corpe et al. 2010).

Studies of *slc23a1*^−/−^ mice highlight the central role of the kidney in maintenance of systemic vitamin C concentrations, via renal reabsorption (Sotiriou et al. 2002; Corpe et al. 2010). Reabsorption of ascorbic acid across the polarized renal epithelial cell requires at least two events: uptake from the tubular lumen across the apical membrane into the tubule cell, followed by release across the basolateral membrane into the circulation. Experiments show that SLC23A1 mediates ascorbate uptake across the apical membrane into the cell (Kuo et al. 2005; Johnston and Laverty 2007; Luo et al. 2008). What is unknown is the mechanism of ascorbic acid efflux from the renal epithelial cell, across the basolateral membrane. High presence of the rat slc23a1 protein was observed in the brush border of the renal proximal tubule (Lee et al. 2006).

Given that heterologous SLC23A1 locates to the apical and basal pole and that heterologous SLC23A2 locates to the basolateral surface in Madin-Darby Canine Kidney Epithelial Cells (MDCK) cells, we hypothesized that either one or both transporters could have a role in basal release. Heterologous green fluorescent protein tagged SLC23A1 is mainly not only located at the apical pole in confluent polarized renal epithelial MDCK cells in culture, but also shows significant localization at the lateral and basal pole (Boyer et al. 2005). SLC23A1 overexpression increases apical but not the basolateral ascorbic acid uptake in MDCK cells (Boyer et al. 2005). In contrast, heterologous green fluorescent protein tagged SLC23A2 localizes exclusively to the basolateral surface of the renal epithelial MDCK cells in culture, and overexpression elevates basal ascorbic acid uptake (Boyer et al. 2005).

There is a lack of adequate experimental models to explore mechanisms of ascorbate efflux in more detail. For this reason, we developed and tested a new *Xenopus laevis* oocyte expression system, and utilized ascorbate transporter overexpression and fine mapping techniques.

## Material and Methods

### Expression analysis in the nephron

#### In silico analysis of SLC23A1 and SLC23A2 gene expression in the human nephron using SAGE libraries

Differentially expressed transporter genes were identified using an *in silico* approach. Previously deposited SAGE libraries (Chabardes-Garonne et al. 2003) constructed from microdissected human kidney samples were downloaded in their entirety from the GEO archive (Barrett et al. 2005). These data were matched as described previously (Velculescu et al. 1995) to Unigene clusters using the annotated *SauIII* 10 bp files available from the NCBI SAGEmap FTP site (Lash et al. 2000). To increase the sensitivity of the screen, multiple tags corresponding to the same Unigene cluster were summed and counted as a single entity using MS Access. The gene expression was then determined by comparing genes differentially expressed in the kidney segments without setting a minimum tag count in all of the libraries.

### Ascorbic acid transport and release in *X. Laevis* oocytes

#### Isolation of *X. laevis* oocytes

Ovaries were resected from adult female frogs anesthetized with 3-aminobenzoic acid ethyl ester (2 g/750 mL) (Sigma-Aldrich, St Louis, MO) in ice water. Ovarian lobes were opened and washed in 2 changes of OR-2 without calcium (5 mmol/L HEPES (2-[4-(2-hydroxyethyl)piperazin-1-yl]ethanesulfonic acid), 82.5 mmol/L NaCl, 2.5 mmol/L KCl, 1 mmol/L MgCl_2_, 1 mmol/L Na_2_HPO_4_, 100 μg/mL gentamicin, pH 7.8) with collagenase (2 mg/mL) (Sigma-Aldrich) for 30 min each at 23 °C. Individual oocytes (stages V and VI) were isolated from connective tissue and vasculature, transferred to calcium-containing OR-2 (1 mmol/L CaCl_2_), and maintained at 18–20°C until injection with cRNA.

#### Complementary RNA production and injection

Isolated oocytes were injected with cRNA coding for the human ascorbic acid transporter SLC23A1 and/or glucose transporter GLUT1. Complementary RNA was prepared by in vitro runoff reverse transcription of linearized plasmids carrying the open reading frame of interest utilizing SP6 or T3 mMessage mMachine (Ambion/Life Technologies, Carlsbad, CA) following the manufacturer's protocol. Oocytes were injected utilizing a Nanoject II injector (Drummond Scientific, Broomall, PA). Injection volumes were 36.8 nL, and cRNA concentrations of 0.5 ng/nL for the single transporter injections and 1 ng/nL for the dual injections. Sham-injected oocytes were injected with 36.8 nL of water. Post injection oocytes were maintained in OR-2 medium containing 1 mmol/L pyruvate (Sigma-Aldrich) at 18–20°C until experiments were performed.

#### Oocyte transport studies

[^14^C]Dehydroascorbic acid was prepared from crystalline [^14^C]ascorbic acid (8.0 mCi/mmol; NEN Life Science Products Inc., Boston, MA). A volume of 5 μL of bromine solution (Fluka/Sigma-Aldrich) was added to 600 μL of [^14^C]ascorbic acid solubilized in ultrapure water at a concentration of 20 mmol/L, vortexed briefly, and immediately purged with nitrogen on ice and in the dark for 10 min. High-performance liquid chromatography (HPLC) analyses compared with scintillation spectrometry confirmed 100% conversion of ascorbate to DHA.

Two days post injection, oocytes were equilibrated at room temperature in OR-2. Experimental oocytes coexpressing SLC23A1 and GLUT1 or controls expressing only GLUT1 were incubated with 1 mmol/L [^14^C] Dehydroascorbic acid in OR-2 for up to 60 min at 23°C. At different time intervals, ascorbic acid in the medium as well as in individual oocytes was determined by HPLC, as described previously (Washko et al. 1993). After incubation, oocytes were washed immediately four times with 4 mL of ice-cold transport buffer. Individual oocytes were either dissolved in 500 μL of 10% sodiumdodecyl sulphate (SDS), and internalized radioactivity was measured using scintillation spectrometry, or 50 μL of 60% methanol (1 mmol/L EDTA [ethylenediaminetetraacetic acid]) was added to one oocyte, followed by centrifugation at 20,000*g* for 15 min. Supernatants were frozen at −70°C for later HPLC analysis.

Ascorbic acid uptake mediated by SLC23A1 was determined by incubating oocytes with different concentrations of [^14^C] ascorbic acid in OR-2. Intracellular [^14^C] ascorbic acid was determined based on internalized radioactivity and/or HPLC, as described in legends.

### Ascorbic acid transport and release in mammalian cells

#### Cell culture and transfection

Chinese Hamsters Ovary cells (CHO) were obtained from ATCC (Manassas, VA). The transfection construct was made by inserting 1797 base pairs of the human *SLC23A1* open reading frame into pcDNA 6/V5-His C vector (Invitrogen, Carlsbad, CA) between *Hin*dIII and *Eco*RI cloning sites. CHO cells were transfected with the construct using LipofectAMINE PLUS kit (Invitrogen). One day before transient transfection, CHO cells growing in Ham's F-12 medium on 60-mm plates were incubated with 0.05% trypsin, EDTA (Invitrogen) for 5 min and counted. 10^7^ cells were re-plated on 60-mm plates and achieved 50–90% confluency in 24 h in Ham's F-12 medium with 10% heat-inactivated fetal calf serum. Cells were then washed once in phosphate-buffered saline, and 2 mL of Ham's F-12 medium without serum were added to each 60-mm plate. To prepare transfection mixtures for each plate, 2 μg of DNA in 2 μL of H_2_O were diluted into 240 μL of medium without serum and 8 μL of AMINE PLUS reagent. In a second tube, 12 μL of LipofectAMINE PLUS reagent was diluted into 238 μL of medium without serum. The two tubes were incubated for 15 min at room temperature, combined, and incubated for an additional 15 min at room temperature. The combined transfection mixture (500 μL) was added to each plate, which was gently swirled and then incubated at 37°C, 5% CO_2_ atmosphere. After 3 h, the medium was replaced with Ham's F-12 medium with 10% fetal bovine serum. Transiently transfected cells were used in experiments within 24–48 h.

#### Mammalian cell transport studies

Transfected CHO cells in 24-well plates were washed once and incubated with Krebs buffer (30 mmol HEPES, 130 mmol NaCl, 4 mmol KH_2_PO_4_, 1 mmol MgSO_4_, 1 mmol CaCl_2_, pH 7.4). Transport was initiated by adding 250 μmol/L DHA. After incubation at 37°C, uptake was stopped by washing cells in ice-cold phosphate-buffered saline at the times specified. Cells were solubilized in phosphate buffered saline (PBS) containing NaOH (0.1 mol/L) and CHAPS (10 g/L; Sigma-Aldrich) and analyzed by scintillation spectrometry or HPLC as described (Washko et al. 1993; Rumsey et al. 2000). To confirm SLC23A1-mediated ascorbic acid uptake, CHO cells were incubated with different concentrations of [^14^C] ascorbic acid in Krebs buffer and intracellular [^14^C] ascorbic acid was determined based on the internalized radioactivity. Intracellular volumes were determined as described before (Washko et al. 1993; Wang et al. 1997).

### Statistics

Data displayed represent mean values ± S.D. of three replicates, and each experiment was repeated a minimum of three times with similar results. Error bars were omitted when the S.D. was less than symbol size.

Data points (each representing the mean value of 10–15 oocytes as above) were analyzed by nonlinear regression analysis using Sigmaplot graphing software package 5.1 (Systat Software Inc., San Jose, CA).

## Results

### Localization of the *SLC23A1* and *SLC23A2* transcripts in the nephron

**Figure 1:**
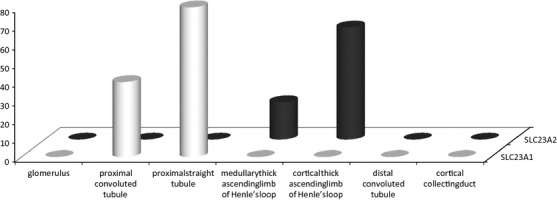
Sites of expression of *SLC23A1* and *SLC23A2* in microdissected segments of the human nephron: *SLC23A1* expression is defined to the proximal convoluted and proximal straight tubule. *SLC23A2* is found in the medullary and cortical thick ascending limb of Henle's loop. Data are depicted as SAGE tags/million tags.

To determine whether SLC23A1 and/or SLC23A2 play a role in ascorbate efflux, their precise locations of expression must be determined in the renal nephron. If messages for both *SLC23A1* and *SLC23A2* colocalize in some renal epithelia, both transporters could mediate ascorbate release. Alternatively, if only one transporter is expressed, then only its role as an ascorbic acid release protein needs to be subsequently tested. To investigate expression, we analyzed human SAGE data for microdissected human kidney segments (GSM10419 and GSM10423-GSM10429) (Chabardes-Garonne et al. 2003). We found very high *SLC23A1* expression in the proximal convoluted and straight tubule (Fig. 1), and no expression in other parts of the nephron. In contrast, *SLC23A2* was not found in the proximal convoluted tubule, but was expressed in both the medullary and the cortical thick ascending limbs of Henle's loop (Fig. 1).

Expression of *SLC23A1* in the proximal convoluted and straight tubule, the major site of sodium-dependent organic solute reabsorption, is consistent with the key role of SLC23A1 in renal ascorbic acid re-absorption, recently demonstrated in the *slc23a1*^*−/−*^ mouse (Corpe et al. 2010). Dependent on its location on the cell membrane, the SLC23A1 protein could have a dual role in renal ascorbic acid reabsorption in the proximal tubular epithelia cell, mediating both the apical uptake from the tubular lumen as well as the basolateral release into the blood capillaries. As an example, in the MDCK cell model of polarized renal tubular epithelial cells, heterologous expressed SLC23A1 protein is mainly localized at the apical brush border, but with a significant basolateral presence (Boyer et al. 2005; Varma et al. 2008).

Because SLC23A1 is necessary for ascorbic acid reabsorption, if SLC23A2 is also involved, the two transporters should colocalize. Both proteins should be present in the same cell type to form a functioning apical uptake to basal release system. As the message for *SLC23A2* does not colocalize with *SLC23A1*, a role of SLC23A2 as the ascorbic acid release protein in the renal proximal tubular epithelia cell can be excluded (Fig. 1). Subsequent experiments were focussed to test whether SLC23A1 could mediate ascorbic acid release.

### Heterologous SLC23A1 in *X. laevis* oocytes

**Figure 2:**
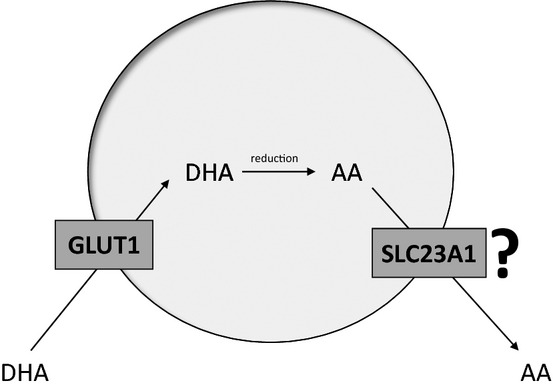
Dual-transporter *Xenopus laevis* oocyte expression system to determine ascorbic acid release. Glucose transporter isoform 1 (GLUT1) and SLC23A1 as a putative protein mediating cellular efflux (Effluxer) are coexpressed in a *X. laevis* oocyte. As a result, dehydroascorbic acid (DHA) is transported into the oocytes using facilitated diffusion via GLUT1, and once intracellular, it is reduced to ascorbic acid (AA). Dehydroascorbic acid is not a substrate of the putative Effluxer, which can utilize the intracellular ascorbic acid as a substrate and release it into the medium.

To explore whether SLC23A1 mediates cellular release of ascorbic acid, we developed a new dual transporter *X. laevis* oocyte assay system in which glucose transporter 1 (GLUT1) and SLC23A1 are coexpressed (Fig. 2). The *X. laevis* oocyte expression system has been successfully used to characterize proteins mediating uptake of ascorbic acid and its oxidized product dehydroascorbic acid (Rumsey et al. 1997, 1999, 2000; Daruwala et al. 1999; Corpe et al. 2013). Dehydroascorbic acid transport is mediated by several facilitated glucose transporter proteins, with highest activity found for GLUT1 and GLUT3 (Rumsey et al. 1997; Corpe et al. 2013). Once dehydroascorbic acid enters cells and *Xenopus* oocytes, it is immediately reduced to ascorbic acid. Ascorbic acid is not a substrate for facilitated glucose transporters (Rumsey et al. 1997, 1999, 2000), and is trapped unless another protein mediating its release is present on the membrane. We utilized these findings in the oocyte expression system by coinjecting cloned RNAs (cRNAs) for GLUT1 and for SLC23A1, a putative ascorbate release transporter. In these oocytes, if expressed SLC23A1 mediated ascorbic acid efflux, then after exposure of ooctyes to dehydroascorbic acid, we predicted that extracellular ascorbate would increase compared to controls without injected cRNA for SLC23A1. Similarly, decreased intracellular ascorbate concentrations would be predicted in oocytes injected with both transporter cRNAS compared to oocytes exposed to dehydroascorbic acid but without coinjection of SLC23SLC23A1 cRNA (Fig. 2).

**Figure 3:**
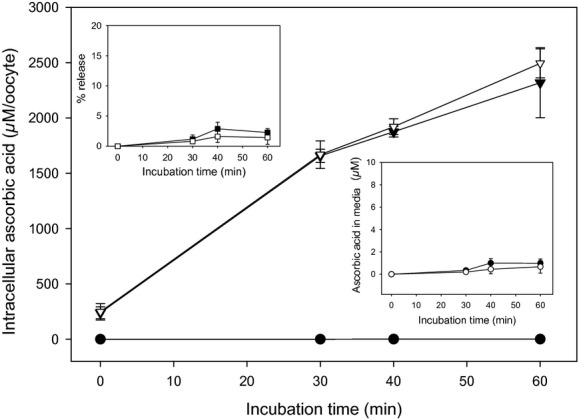
SLC23A1 does not mediate ascorbic acid efflux in *Xenopus laevis* oocytes: Oocytes expressing GLUT1 (∇) only or coexpressing SLC23A1 + GLUT1 (▼) were incubated with 1 mmol/L dehydroascorbic acid, resulting in intracellular ascorbic acid concentrations of up to 2.5 mmol/L/oocyte. Sham-injected oocytes did not transport dehydroascorbic acid and therefore did not accumulate intracellular ascorbic acid (•). *Inset right bottom:* Ascorbic acid concentration in incubation medium of oocytes expressing GLUT1 (•) or coexpressing SLC23A1 + GLUT1 (○). *Inset left top:* relative ascorbic acid release in% of oocytes expressing GLUT1 (▪) or coexpressing SLC23A1 + GLUT1 (□). Intracellular [^14^C]ascorbic acid was determined based on internalized radioactivity and/or HPLC coupled with electrochemical detection.

Using the new assay system, GLUT1 alone or GLUT1 and SLC23A1 cRNAs were coinjected into *X. laevis* oocytes, which were then exposed to dehydroascorbic acid. GLUT1 expression alone or combined with SLC23A1 produced oocyte ascorbate concentrations for efflux as high as 2.5 mmol/L. However, for both conditions the concentration of ascorbic acid in the incubation buffer never exceeded 1 μmol/L (Fig. 3 right inset), and the percentage of ascorbic acid in the medium was always below 2.5 % of the intracellular amount (Fig. 3 left inset). The presence of SLC23A1 did not enhance intracellular ascorbic acid concentration, depletion, or the amount detected in the incubation buffer compared to controls without SLC23A1.

**Figure 4:**
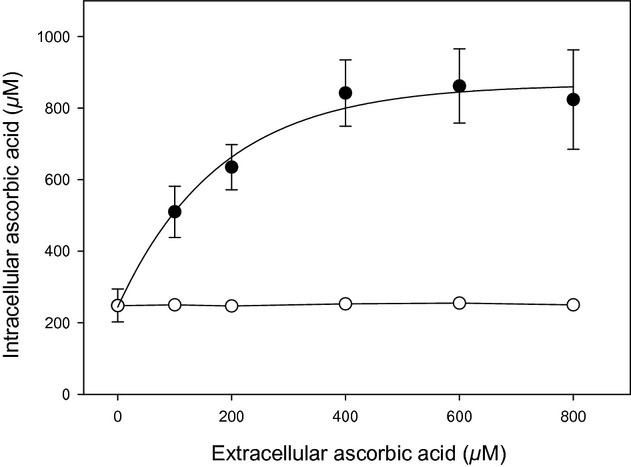
Concentration-dependent ascorbic acid uptake in SLC23A1 injected oocytes. SLC23A1 expressing (•) and sham-injected oocytes (○) were incubated with [^14^C]ascorbic acid (AA) in concentrations from 0 to 800 μmol/L and elevated uptake in the SLC23A1 expressing (•) oocytes demonstrates the presence of the functional protein. Intracellular [^14^C]ascorbic acid was determined based on internalized radioactivity, for oocytes incubated for 10 min.

When SLC23A1 is co-injected with GLUT1 into *X. laevis* oocytes, the data show that both transporters are expressed and functional, and do not interfere with each other. This is demonstrated by two observations: (1) GLUT1-mediated intracellular ascorbic acid accumulation in the coinjected oocytes is identical compared to only GLUT1-injected oocytes when incubated with the same amount of dehydroascorbic acid (Fig. 3); and (2) SLC23A1 mediates sodium-dependent ascorbic acid uptake into the coinjected oocytes with kinetics similar to those previously reported (Fig. 4) (Daruwala et al. 1999; Boyer et al. 2005).

These experiments indicate that SLC23A1 mediates cellular uptake of ascorbic acid, but not its release from *X. laevis* oocytes. Although the *X. laevis* oocyte expression system is useful for studying many membrane transporters (Sobczak et al. 2010), it remains possible that there could be differences in transcription and/or posttranslational modification in amphibian cells compared to mammalian cells. Therefore, we explored efflux further using mammalian cells.

### Heterologous SLC23A1 in Chinese hamsters ovary cells

**Figure 5:**
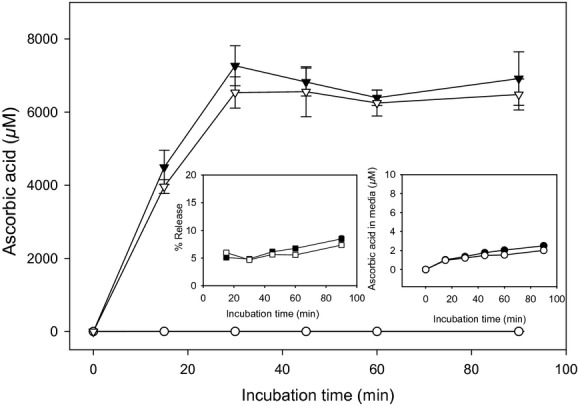
SLC23A1 does not mediate ascorbic acid efflux in Chinese Hamsters Ovarian (CHO) cells. CHO cells expressing SLC23A1 (▼) or vector only (▽) were incubated with 250 μmol/L dehydroascorbic acid, resulting in rapid elevations of intracellular ascorbic acid up to a concentration of 7 mmol/L. CHO cells do not contain any detectable ascorbic acid (○). *Inset right bottom*: ascorbic acid concentration in incubation medium of SLC23A1 overexpressing CHO cells (•) and vector alone containing CHO cells (○). *Inset centre bottom:* relative ascorbic acid release in% of SLC23A1 overexpressing CHO cells (▪) and vector alone containing CHO cells (□). Ascorbic acid was determined using HPLC coupled with electrochemical detection.

CHO mammalian cells overexpressing SLC23A1were compared to vector controls to investigate whether SLC23A1 mediates ascorbate release. Overexpressing cells and controls were loaded with ascorbic acid by incubating them with dehydroascorbic acid, which is transported on endogenous GLUT transporters and immediately reduced internally to ascorbic acid. A rapid increase in intracellular ascorbic acid occurred within the first 30 min of dehydroascorbic acid incubation (Fig. 5). Peak concentrations for subsequent efflux were approximately 7 mmol/L after 30 min, and 7 mmol/L concentrations were maintained for the following 60 min (Fig. 5). However, concentrations in the incubation medium for both overexpressing and control cells were more than 1000-fold lower, and never surpassed 3 μmol/L (Figure 5 right inset). Similarly, percentage of total cell ascorbate that underwent efflux was similar whether or not SLC23A1 was overexpressed (Fig. 5 left inset).

**Figure 6:**
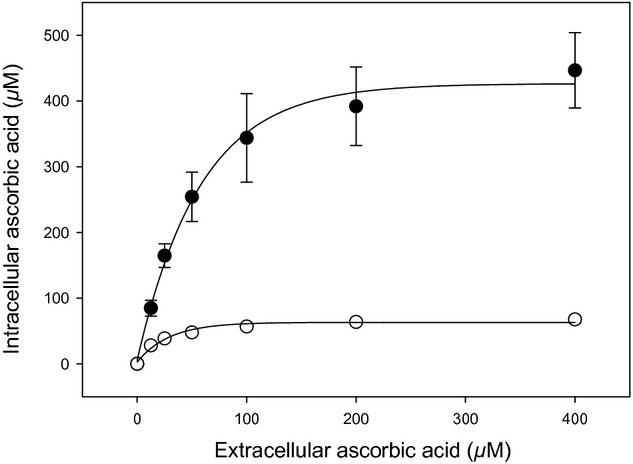
Concentration-dependent ascorbic acid uptake in SLC23A1-transfected CHO cells. SLC23A1 overexpressing CHO cells (•) and vector alone containing CHO cells (○) were incubated with ascorbic acid 0–400 μmol/L. Elevated uptake in the SLC23A1 expressing (•) oocytes demonstrates the presence of the functional protein. Ascorbic acid was determined using HPLC coupled with electrochemical detection, for cells incubated for 10 min.

Wild-type CHO cells do not express SLC23A1 (Song et al. 2002). Transient transfection resulted in increased ascorbic acid uptake, demonstrating the presence of the functional transporter (Fig. 6). In CHO cells transfected to overexpress SLC23A1, ascorbic acid uptake followed saturation kinetics and exceeded the uptake in wild-type cells by as much as 10-fold, confirming the presence of the functional protein (Fig. 6). Despite being a functional transporter, because ascorbic acid efflux from SLC23A1-transfected CHO cells was not elevated in comparison to nontransfected cells (Fig. 5), it can be concluded that SLC23A1 does not mediate ascorbic acid release in this mammalian cell model.

## Discussion

The data here show that neither sodium-dependent ascorbic acid transporters SLC23A1 nor SLC23A2 function as the elusive ascorbic acid release protein on the basolateral side of the renal epithelial cell. Regarding SLC23A1, it is located in the renal proximal tubular epithelial cell, the main site of renal reabsorption for small solutes like ascorbic acid. Our studies showed that heterologous expressed functional human SLC23A1 did not elevate ascorbic acid release in *X. laevis* oocytes and CHO cells. These results suggest that SLC23A1 does not have a role in mediating ascorbic acid release in renal epithelial cells, and by extrapolation to other epithelial cell types. Regarding SLC23A2, because it is not expressed in the renal proximal epithelial cell, and therefore does not colocalize with *SLC23A1* in the renal proximal tubular epithelium, SLC23A2 can be excluded from participating in ascorbate release in the proximal renal epithelial cell. However, we do not rule out the possibility of a role for SLC23A2 in cellular ascorbic acid release in other epithelial tissues, including extrarenal tissues (Boyer et al. 2005). Ascorbic acid release has been observed in a variety of extrarenal cell types (Upston et al. 1999), including hepatocytes, glutamate stimulated astrocytes (Korcok et al. 2000; Wilson and Dragan 2005), stimulated coronary artery endothelial cells (Davis et al. 2006a,b), and intestinal carcinoma cells (unpublished observation). In the enterocyte, the message for *SLC23A2* is present. Thus, its possible role in cellular ascorbic acid release from extrarenal tissues cannot be ruled out (Boyer et al. 2005). Further studies are needed to determine whether SLC23A2 plays a role in ascorbate efflux in extrarenal tissues.

It has been proposed that the membrane proteins responsible for cellular ascorbic acid release are distinct from the glucose/dehydroascorbic acid (GLUT) transporters (Upston et al. 1999). This is based on the observation that in all experiments investigating cellular release, ascorbic acid but not dehydroascorbic acid was detected in the extracellular medium (Upston et al. 1999). These data are also consistent with detection of ascorbic acid only in the cell cytosol. Thus, we suggest that GLUT-type membrane transporter proteins do not participate in basolateral release of ascorbic acid in epithelial cells.

The presented data also allow us to conclude that SLC23A1 exclusively mediates apical ascorbic acid uptake in the proximal tubular epithelia cell. We recently demonstrated that *Slc23a1* in the mouse has indispensible roles in renal ascorbic acid reabsorption and systemic control of ascorbate concentrations (Corpe et al. 2010). We show here that SLC23A1 is localized exclusively to the proximal tubule. The findings are consistent with data concerning the role of human SLC23A1in apical ascorbic acid transport into polarized renal epithelial cells (Boyer et al. 2005). Considering these reports, plus our findings that SLC23A1 does not mediate ascorbic acid release, we conclude that SLC23A1 plays a nonredundant function in renal ascorbic acid reabsorption.

Understanding the mechanism of basolateral release of ascorbic acid is as important as identifying the mechanism of apical uptake, due to the potential detrimental impact of variations on genes responsible for both functions. Function-changing variations could elevate the risk for disease development due to suboptimal systemic ascorbic acid levels, as seen in the *slc23a1*^−/−^ mouse studies. Thus, even at recommended daily intake levels some humans may receive suboptimal ascorbic acid levels to maintain health, if there is a function-changing variant on the gene(s) responsible for renal reabsorption (Timpson et al. 2010). Because appropriate levels of ascorbic acid may be coupled to maintaining health and preventing disease, it is worthwhile to continue to identify the genes and proteins responsible for ascorbate efflux from epithelial cells in the kidney and intestine.

We describe here a novel dual transporter system utilizing *X. laevis* oocytes to investigate the function of putative ascorbic acid release proteins. The data show that SLC23A1 and GLUT1 are expressed and fully functional when their cRNAs coinjected into *X. laevis* oocytes. SLC23A1 and GLUT1 do not interfere with each other, confirming the utility of the system to characterize putative ascorbic acid release proteins. A fundamental characteristic of the oocyte system is that extracellular dehydroascorbic acid is used to increase intraoocyte ascorbate concentrations. SLC23A1 cannot mediate uptake because extracellular dehydroascorbic acid is not its substrate (Daruwala et al. 1999). Extracellular dehydroascorbic acid is taken up via GLUT1, and upon entry immediately reduced to ascorbic acid (Fig. 3) (Rumsey et al. 1997, 1999, 2000; Corpe et al. 2005, 2013). The intracellular ascorbate is no longer a GLUT1 substrate, but would now be available for possible release via SLC23A1 (or any other coinjected transporter). Using dehydroascorbic acid loading via GLUT1, we were able to boost internal ascorbic acid concentrations as high as 2500 μmol/L, which are above the threshold of activity for the putative ascorbic acid release protein (Levine et al. 1999; Upston et al. 1999; Korcok et al. 2000; Wilson and Dragan 2005; Davis et al. 2006a,b). If SLC23A1-mediated release, we therefore should have been able to detect it. The lack of release function for SLC23A1 was also confirmed in overexpressing CHO cells.

Overall, the presented data prove that neither SLC23A2 nor SLC23A1 is involved in the cellular ascorbic acid release in the renal proximal tubular epithelial cell. In addition, we describe SLC23A1's key role in apical uptake in the renal epithelial cell. Due to this nonredundant function in renal ascorbic acid reabsorption, any genetic variation decreasing SLC23A1 transport capacity will impact an individual's ability to retain vitamin C, which could ultimately lead to suboptimal ascorbate levels in these individuals even at recommended adequate intake levels. Suboptimal ascorbate levels are associated with elevated risks of common diseases (Padayatty et al. 2003). Therefore, we believe it is warranted to test the impact of other nonsynonymous genetic variations in the *SLC23A1* gene on transport function. We also believe it is warranted to identify basolateral ascorbate release protein(s), as genetic variations in these might also negatively impact the ability to reabsorb ascorbic acid.
